# Editorial: Oxidants and Redox Signaling in Inflammation

**DOI:** 10.3389/fimmu.2019.00545

**Published:** 2019-03-26

**Authors:** Bhupesh Singla, Rikard Holmdahl, Gabor Csanyi

**Affiliations:** ^1^Vascular Biology Center, Medical College of Georgia, Augusta University, Augusta, GA, United States; ^2^Section for Medical Inflammation Research, Department of Medical Biochemistry and Biophysics, Karolinska Institutet, Stockholm, Sweden; ^3^Center for Medical Inflammation Research, Southern Medical University, Guangzhou, China; ^4^Department of Pharmacology and Toxicology, Medical College of Georgia, Augusta University, Augusta, GA, United States

**Keywords:** reactive oxygen species, inflammation, NADPH oxidase, NCF1, PKCδ, Nrf2, Hsp70

Inflammation is a biological response of the host to any harmful (infectious or sterile) aberration. Anti-inflammatory and pro-resolving mediators are secreted shortly after the beginning of the inflammatory response to revert destruction and repair tissues. Inadequate transition from the inflammatory phase to resolution could lead to a plethora of chronic inflammatory diseases, including atherosclerosis, cancer, chronic respiratory disorders, arthritis, inflammatory bowel disease, allergies, and Alzheimer's disease (1, 2).

During the last two decades, a shift in the view of the role of reactive oxygen species (ROS) has emerged to be viewed as critical regulators of the inflammatory process. ROS were previously viewed mainly as driving inflammation through toxic effects, based on their strong association with the inflammatory response and direct toxic effects at high concentrations (3). However, genetic evidence underlined a much broader role of ROS, as it was found that experimental animals with genetic variants leading to low ROS responsiveness have a higher risk of developing autoimmune diseases (4–6). More recently, it was shown that Ncf1 (p47^*phox*^) and low ROS responsiveness are important factors in human autoimmune diseases. As a direct follow up of the experimental studies, a single nucleotide polymorphism in the *Ncf1* gene was recently shown to be an important genetic factor in the common autoimmune disease systemic lupus erythematosus (SLE) (6, 7).

The biological effects of ROS are complex and regulate the inflammatory response in specific ways. ROS regulate intracellular signaling by oxidizing amino acid residues, such as cysteine and lysine, oxidize lipids and nucleic acids, and under physiological conditions are kept in balance and compartmentalized by antioxidant enzymes (8, 9). Despite great progress in the field of redox biology, much remains unknown regarding the specific redox mechanisms that control the inflammatory process and mediate the pathogenesis of chronic inflammatory disorders.

This Research Topic entitled “*Oxidants and Redox Signaling in Inflammation*” focuses on the sources of ROS in inflammation, further explores how ROS interact with their downstream targets, identifies novel redox cell signaling pathways, discusses new ROS detection techniques and describe new therapeutic strategies to treat chronic inflammatory diseases. The published research articles are briefly described below.

The review article published by Reshetnikov et al. introduces the pivotal role of neutrophils and neutrophil-derived ROS in innate immune response and regulation of inflammatory responses involving cytotoxic effects, neutrophil extracellular trap formation, secretion of various proteases and antimicrobial factors. They highlight various compounds which can amplify ROS production by neutrophils both *in vitro* and *in vivo* and discuss their therapeutic potential. In addition, they summarize possible approaches to enhance ROS production to treat inflammatory, autoimmune, and bacterial diseases. The review article by Sirokmány and Geiszt provides a timely overview of heme peroxidases and their associations with H_2_O_2_-generating NADPH oxidases. Using human and metazoan examples, the authors describe the functional relationship and biological consequences of the tandem interaction between NADPH oxidases and heme peroxidases.

Nuclear factor erythroid 2-related factor 2 (Nrf2) is a transcription factor that provides resistance to oxidative stress and metal toxicity (10). Encountering ROS, Nrf2 binds to the antioxidant response elements located in the upstream promoter region of antioxidant genes and initiates their transcription. The first original research article in this issue by Han et al. investigated the therapeutic potential of dimethyl fumarate (DMF), an Nrf2 activator, for the treatment of acute graft-vs.-host disease (aGVHD). The authors report that DMF treatment attenuated histological damage and improved the survival of mice with aGVHD following MHC-mismatched allogeneic hematopoietic stem cell transplantation (allo-HSCT). Another study by Ohl et al. using murine models evaluated the effects of constitutive Nrf2 activation on myeloid-derived suppressor cells (MDSCs), which are known regulators of pathological immune responses due to their potent immunosuppressive effect. They demonstrated that constitutive Nrf2 activation in hematopoietic cells (*VAV*^*cre*^*Keap*^*fl*/*fl*^) upregulates immunosuppressive MDSCs and maintains metabolic homeostasis of these cells. In addition, mice with constitutive Nrf2 activation were resistant to lethal doses of LPS, indicating a key role of Nrf2 in the generation of tolerant MDSCs and protection against sepsis.

NADPH oxidases (Nox) are multi-subunit enzyme complexes that transfer electrons to oxygen to generate superoxide anion (${\text{O}}_{2}^{•-}$), or its dismuted form, hydrogen peroxide (H_2_O_2_) (11). Nox2 is dormant in quiescent cells and becomes rapidly activated upon exposure to various exogenous stimuli. Singla et al. found that activation of PKCδ and Nox2-derived ROS stimulate antigen macropinocytosis in immature dendritic cells (iDCs). Using genetically modified iDCs, they demonstrated the role of PKCδ-mediated Nox2 activation in iDC macropinocytosis, maturation and subsequent secretion of T-cell stimulatory cytokines, which are crucial for T-cell mediated immune responses. Chronic granulomatous disease (CGD) is characterized by mutations in the *CYBB* gene (encodes Nox2) and CGD patients are more susceptible to pathogenic infection due to decreased ROS generation (12). The interesting study published by Cachat et al. showed elevated levels of IgG_2_ in both CGD patients and Nox2 knockout mice, increased IgG_1_, IgG_2b_, and IgG_2c_ production following immunization with ovalbumin + curdlan (dectin-1 agonist) and augmented T-cell activation in Nox2-deficient mice. Following stimulation of Nox2-deficient DCs, they found an increase in the release of Th_1_ stimulating cytokines. The authors concluded that DC Nox2 plays an important role at the interface of innate and specific immunity.

Nox1 is a major source of ROS in the colon (13). Nox1 consists of the membrane-bound p22^*phox*^ (*CYBA*) and two cytosolic subunits (NoxA1 and NoxO1). As NoxO1 lacks the autoinhibitory region (AIR) observed in Ncf1, NoxO1 is able to associate with p22^*phox*^ constitutively, leading to ${\text{O}}_{2}^{•-}$ generation in the absence of exogenous stimuli. The study by Moll et al. demonstrated that lack of NoxO1 stimulates proliferation and inhibits apoptosis of colon epithelial cells. Furthermore, NoxO1 deletion increased the severity of dextran sulfate sodium (DSS)-induced colitis and contributed to the development of azoxymethane/DSS-induced colon cancer. An original research investigation by Carvalho et al. studied DSS-induced acute and chronic colitis in wild type and Ncf1-mutant mice. They observed more severe clinical scores of colitis in mutant mice compared to controls. Interestingly, Ncf1-mutant, but not control, mice developed adenocarcinoma in the DSS-induced colitis model. The authors concluded that Ncf1-mediated ROS generation is essential to prevent the development of adenocarcinoma from chronic colitis.

Proniewski et al. employed a novel technique using immuno-spin trapping and fluorescent detection of DMPO nitrone adducts to characterize and quantify the progression of oxidative modifications in a murine model of heart failure (Tgαq^*^44 mice). Progressive elevation of DMPO nitrone adducts was detected in the cardiomyocytes and coronary endothelium of 10- to 16-month-old Tgαq^*^44 mice compared to age-matched controls.

Pneumonia is a leading cause of death in children and elder individuals worldwide (14, 15). However, the mechanisms by which pneumolysin (PLY), a toxin produced by causative bacteria, induce pulmonary permeability edema and acute lung injury are not fully understood. Li et al. have shown that Hsp70 overexpression using adenovirus-mediated gene transfer or geranylgeranylacetone (GGA) attenuate PLY-induced increase in lung microvascular endothelial cell permeability via reducing mitochondrial ROS production and cell death. These results suggest that acute upregulation of Hsp70 may be an effective therapeutic approach in the treatment of lung injury associated with pneumonia.

Finally, Widdrington et al. investigated the association between compensatory immune responses and mitochondrial function, triggered by an inflammatory stimulus in monocytes. Exposure of monocytes to LPS caused early oxidative stress (6 h), which was resolved by induction of antioxidant mechanisms and mitochondrial degradation through mitophagy.

The articles published in this Research Topic contribute to a better understanding of redox regulation of inflammatory processes. The knowledge gained from these studies may help identifying novel therapeutic targets to aid resolution and combat chronic inflammatory disorders.

## Author Contributions

All authors contributed to the writing and editing of the editorial and approved it for publication.

### Conflict of Interest Statement

The authors declare that the research was conducted in the absence of any commercial or financial relationships that could be construed as a potential conflict of interest.
